# Anterior percutaneous full-endoscopic transcorporeal decompression of the spinal cord via one vertebra with two bony channels for adjacent two-segment cervical spondylotic myelopathy: a technical note

**DOI:** 10.1186/s12891-023-06978-7

**Published:** 2023-10-26

**Authors:** Wen He, Qian Du, Zheng-Ji Wang, Zhi-Jun Xin, Fu-Jun Wu, Wei-Jun Kong, Heng Su, Wen-Bo Liao

**Affiliations:** 1 Department of Orthopedic Surgery, The Second Affiliated Hospital of Zunyi Medical University, Zunyi, Guizhou China; 2 Department of Spinal Surgery, The Affiliated Hospital of Zunyi Medical University, Zunyi, Guizhou China

**Keywords:** Cervical spondylotic myelopathy, Decompression of spinal cord, Full-endoscopic, Anterior approach, Transcorporeal, Two bony channels.

## Abstract

**Background:**

The current treatments for adjacent two-segment cervical spondylotic myelopathy (CSM) include two-segment anterior cervical discectomy and fusion (ACDF) and single-segment anterior cervical corpectomy and fusion (ACCF). Long-term follow-up has demonstrated that both procedures have complications such as reduced cervical mobility, accelerated degeneration of adjacent segments and loosening of internal fixation screws. The purpose of this study is to demonstrate the feasibility, safety, and efficacy of anterior percutaneous full-endoscopic transcorporeal decompression of the spinal cord (APFETDSC) via one vertebra with two bony channels for the treatment of adjacent two-segment CSM and to present our surgical experience.

**Methods:**

Anterior percutaneous full-endoscopic transcorporeal decompression of the spinal cord (APFETDSC) via one vertebra with two bony channels was performed for 12 patients with adjacent two-segment CSM with follow-up care for at least 12 months. The Visual analog scale (VAS) and the Japanese Orthopedic Association Score (JOA) were recorded, and modified Macnab criteria were used to evaluate the treatment excellence rate. Radiological examinations, including X-ray, computed tomography (CT) and magnetic resonance imaging (MRI), were used to evaluate spinal cord decompression, intervertebral stability and healing of the bony channel.

**Results:**

All 12 patients completed the operation successfully. No postoperative complications, such as dysphagia, Horner’s syndrome, or laryngeal recurrent nerve palsy, were found. The postoperative VAS and JOA scores were significantly improved compared with those before surgery(*P* < 0.001). According to the modified Macnab criteria, the clinical outcome was excellent in 8 cases, good in 3 cases and fine in 1 case at the final follow-up and the excellent and good rate was 91.7%. Postoperative and follow-up imaging showed significant spinal cord decompression, well-healed bony channels and no cervical instability.

**Conclusions:**

This study is the first report of anterior percutaneous full-endoscopic transcorporeal decompression of the spinal cord via one vertebra with two bony channels. This procedure has the advantages of less trauma, faster recovery, fewer complications and no need to implant internal fixators. This is a minimally invasive, feasible and safe surgical procedure for patients with adjacent two-segment CSM.

## Background

Cervical spondylotic myelopathy (CSM) involves a series of signs and symptoms caused by degenerative changes in the cervical spine that compress the spinal cord or its supplying blood vessels. Patients often present with numbness and pain in the extremities, stiffness of movement, a sensation of tied chest and a feeling of stepping on cotton in both feet and, in severe cases, difficulty with urination and defecation [1, 2]. Adjacent two-segment CSM refers to the presence of two adjacent segments with disc herniation, osteophytes at the posterior edge of the vertebral body or ossification of the posterior longitudinal ligament, resulting in spinal cord dysfunction due to compression of the cervical spinal cord in the two adjacent segments. In recent years, as the population ages, the occurrence of adjacent two-segment CSM has become more common, accounting for approximately 56% of CSM cases, and early surgery is mostly recommended due to direct compression of the cervical spinal cord [3, 4].

For the surgical treatment of adjacent two-segment CSM, two-segment anterior cervical discectomy and fusion (ACDF) or single-segment anterior cervical corpectomy and fusion (ACCF) are usually used in clinical practice, both of which enable effective decompression of the spinal cord and maintenance of the height of the fused segment. However, long-term follow-up has demonstrated that these two procedures are associated with complications such as reduced cervical mobility, accelerated degeneration of adjacent segments, and loosening of internal fixation screws [5, 6].

In recent years, spinal endoscopy has developed rapidly, and the anterior transcorporeal approach has been gradually applied in the treatment of cervical degenerative diseases due to its advantages of less trauma, clear visualization, and rapid postoperative recovery [7–11]. Endoscopic surgery for CSM requires not only the removal of herniated disc tissue but also the removal of osteophytes that compress the spinal cord. Kong et al. [12] first reported a 2-year follow-up study of anterior percutaneous full-endoscopic transcorporeal decompression of the spinal cord (APFETDSC) for single-segment CSM in 2019, in which the spinal cord was adequately decompressed and all upper extremity pain and numbness were recovered; no significant surgical complications were found during the follow-up period. Subsequently, Ma et al. [13] conducted a retrospective study of 28 patients with single-segment CSM, all of whom achieved better spinal cord decompression. Follow-up imaging results showed good cervical spine mobility and no occurrence of cervical instability. For adjacent two-segment CSM, more spinal cord is compressed and the clinical symptoms will be more severe, which will inevitably cause more surgical trauma to the patients if open surgery is used. Based on the success of transcorporeal approach spinal cord decompression, we now report the first-ever application of APFETDSC via one vertebra with two bony channels in 12 patients with adjacent two-segment CSM. The purpose of this study was to demonstrate the feasibility, safety, and efficacy of anterior percutaneous full-endoscopic transcorporeal decompression of the spinal cord (APFETDSC) via one vertebra with two bony channels and to provide a new supplementary surgical option for patients with adjacent two-segment CSM.

## Methods

### General information

The study was conducted with the permission of the local Medical Ethics Review Board and informed consent of the patient. In this study, APFETDSC via one vertebra with two bony channels was performed in 12 patients with adjacent two-segment CSM from September 2021 to June 2022, including 7 males and 5 females, aged 48.58 ± 5.02 years (40–56 years), with a symptom duration of 17.75 ± 5.41 weeks (9–30 weeks). The Visual analog scale (VAS) and the Japanese Orthopedic Association Score (JOA) were recorded to evaluate preoperative and postoperative clinical symptoms, and modified Macnab criteria were used to evaluate the treatment excellence rate at the final follow-up.

 The inclusion criteria were as follows: (1) imaging suggestive of compression of the ventral cervical spinal cord at two adjacent segments; (2) clinical symptoms consistent with imaging; (3) progressive worsening of spinal nerve injury symptoms such as limb pain, numbness, weakness or somatic discomfort; (4) ineffective conservative treatment for more than 6 weeks; and (5) no less than 12 months of follow-up. The exclusion criteria were as follows: (1) history of previous surgery in the responsible segment; (2) combined spinal cord tumor, infection or intramedullary lesion; (3) severe osteoporosis; and (4) cervical instability and severe posterior longitudinal ligament calcification.

### Preoperative preparation

Preoperative examinations such as routine biochemical tests, cervical spine X-ray, CT and MRI are completed for patients. Cervical X-ray can be used to evaluate the stability of the vertebral body. CT images can clarify whether there is osteophyte or ligament ossification. MRI is used to determine the location and extent of disc herniation and the degree of spinal cord compression. By combining the above results, the surgeon can more accurately determine the direction and depth of the bony channel. In addition, preoperative insertion of a gastric tube is required to facilitate intraoperative contrast injection, to clarify the esophageal deflection under C-arm fluoroscopy and to determine the direction of the surgical approach. Preoperative training of the trachea and esophagus is also required to avoid postoperative laryngeal spasm and edema.

### Endoscopic instruments

The spinal endoscopy system (SPINENDOS GmbH., Munich, Germany) comprised a 4.3 mm working channel, an outer sheath with a 6.9 mm diameter, a 30°-angled scope with a continuous water irrigation system, a trephine with a 6.5 mm inner diameter and a 7.5 mm outer diameter, a Gimmi-SPINENDOS digital camera system and a low temperature radiofrequency ablation system (ArthroCare Co., Sunnyvale, California, USA). The drill was made by NOUVAG AG (High speed burrs, Goldach, Switzerland).

### Statistical analyses

Statistical analyses were performed using SPSS 29.0 software (IBM Corp., Armonk, NY, USA, Version 29.0) and expressed as mean ± standard deviation. The Shapiro-Wilk method (W-test) was used to check the normality of the data. Paired t-test was used to compare preoperative scores and scores during follow-up, with α = 0.05 as the test level, and *P* < 0.05 was considered statistically significant.

### Surgical technique

**Fig. 1:**
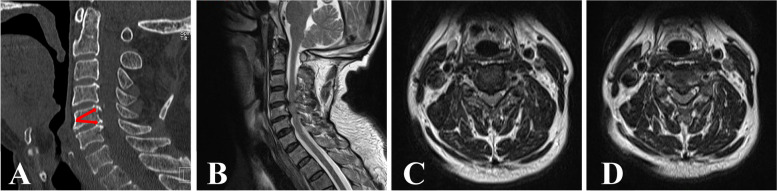
A 48-year-old male was examined for adjacent two-segment CSM. Preoperative sagittal CT image showed severe osteophytes posterior to C4-5 and C5-6 (**A**). The two red arrows in the figure indicate the trajectory of the intended bone channel. Preoperative MRI sagittal images showed that the disc-osteophyte complex in C4-5 and C5-6 significantly compressed the spinal cord (**B**). Preoperative MRI axial image of C4-5 (**C**) and preoperative MRI axial image of C5-6 (**D**)

**Fig. 2:**
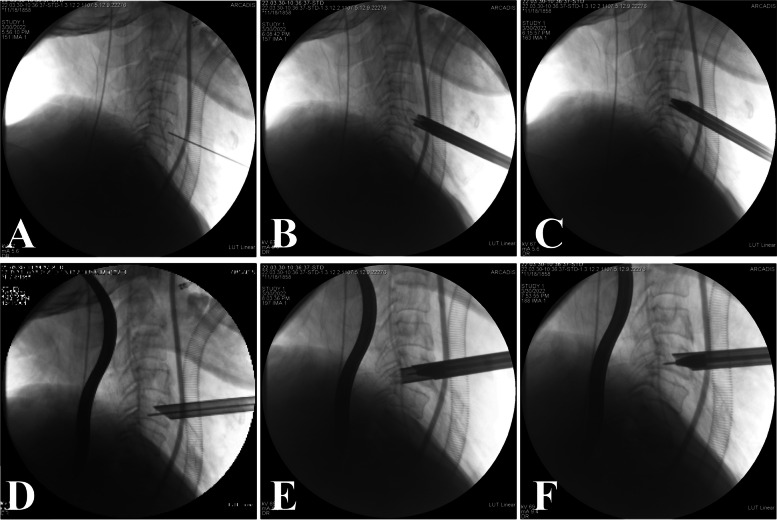
The K-wire was inserted and placed on the anterior surface of C5 under C-arm fluoroscopy (**A**). The trephine was used to establish the bony channel of C4-5 and the direction and depth of the trephine were adjusted under C-arm fluoroscopy (**B**). The working trocar of C4-5 was installed along the dilator (**C**). The working trocar was withdrawn to the entrance of the bony channel and the K-wire was repositioned toward C5-6 under C-arm fluoroscopy (**D**). The trephine was used to establish the bony channel of C5-6 under C-arm fluoroscopy (**E**). The working trocar of C5-6 was installed along the dilator (**F**)

**Fig. 3:**
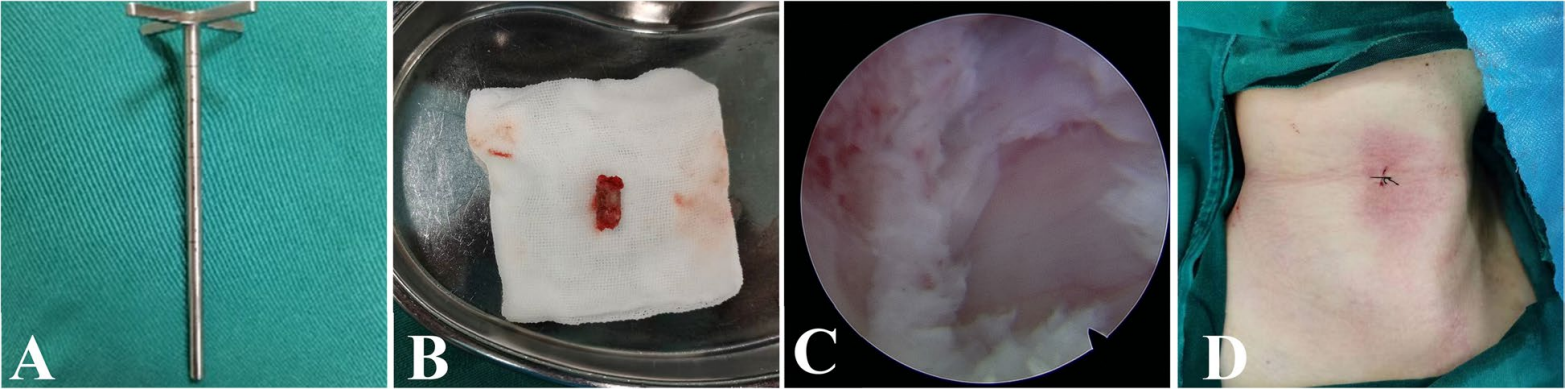
External view of trephine with 6.5 mm inner diameter and 7.5 mm outer diameter (**A**). The bone strip was truncated and protected by saline gauze wrapping (**B**). Good dural pulsation was obtained after removal of the osteophyte and herniated disc tissue (**C**). The incision is closed with one stitch (**D**)

The surgical technique was performed in a patient with C4/5 and C5/6 CSM as a demonstration of the procedure (Fig. 1A-D). The bony channel trajectories are shown in Fig. 1A with two "red arrows". The patient was intubated with general anesthesia, placed in the supine position with shoulder padding of approximately 4 cm, and 20 mL of iohexol contrast agent was injected through the gastric tube. The esophagus was seen to deviate to the left under C-arm fluoroscopy, so the right side of the neck was chosen for the approach. The patient's head was fixed in a slightly hyperextended position and deflected to the esophageal side, and a skin line was drawn at the level of the C5 vertebral body to mark the incision location. Using the "two-finger method" to separate the carotid artery from the esophagus and trachea, the Kirschner wire (K-wire) was positioned at the anterior midpoint of the C5 vertebral body, and the K-wire position was continuously adjusted under C-arm fluoroscopy (Fig. 2A). When the K-wire reached the C4-5 disc-osteophyte complex, the skin was cut approximately 7 mm, and a dilator was placed along the K-wire for blunt separation of the important tissues in the operating area. The dilator was then removed, the trephine (Fig. 3A) was placed along the K-wire, and the vertebral bony channel was established under C-arm fluoroscopy using a rotary cut of the trephine (Fig. 2B). When the trephine reached the C4-5 disc-osteophyte complex, the trephine was slightly rotated and shaken to cut off the vertebral bone strip (Fig. 3B), a working trocar was installed (Fig. 2C), the endoscopic operating system was placed, and a continuous water irrigation system was used intraoperatively to maintain a clear view. The surgeon used a high-speed burr and rongeur to grind the osteophyte at the posterior superior edge of the C5 vertebral body, different types of medullary forceps to remove the herniated disc tissue and part of the posterior longitudinal ligament to obtain good dural pulsation (Fig. 3C), spinal cord decompression, and radiofrequency ablation to stop bleeding in the posterior longitudinal ligament or epidural hemorrhage. When posterior longitudinal ligament or epidural bleeding occurred, a radiofrequency ablation system was used to stop the bleeding. The operating system was then withdrawn to the entrance of the bony channel anterior to the C5 vertebral body, the K-wire was then positioned anteriorly and medially in the C5 vertebral body, and the K-wire position and orientation were adjusted under C-arm fluoroscopy (Fig. 2D). When the trephine reached the C5-6 disc-osteophyte complex, a dilator was placed along the K-wire to bluntly separate the important tissues in the area of operation. After removing the dilator and then placing the trephine along the K-wire, the target bone channel was established using the trephine under C-arm fluoroscopy (Fig. 2E), and when the trephine reached the C5-6 disc-osteophyte complex, the trephine was slightly rotated and shaken to cut off the vertebral bone strip. Next, the working trocar was installed (Fig. 2F). Similar to the procedure in the previous segment, the osteophyte at the posterior inferior margin of the C5 vertebral body was polished using a high-speed burr and rongeur, the herniated disc tissue and part of the posterior longitudinal ligament were removed with medullary forceps, and good dural pulsation and complete decompression were observed. Finally, the previously intercepted autologous bone was trimmed and implanted back into the bony channel, the vertebral canal and bony channel were checked for active bleeding, and the endoscopic operating system was removed. The incision was carefully checked for active bleeding, the incision was closed with one stitch (Fig. 3D), and the sterile material was wrapped and fixed without implanting a drainage tube.

### Postoperative management and follow-up

After surgery, the patients were closely observed for the occurrence of cervical hematoma formation, respiratory distress and asphyxia and were routinely given symptomatic treatment such as decongestion and pain relief. The patients were required to wear a cervical brace for 3 weeks after discharge and take oral mecobalamins (0.5 mg once, three times a day) and multivitamin B (a compound preparation, two tablets at a time, three times a day) for 4–6 weeks to improve neurological function, and they returned to the hospital for follow-up examinations at 3, 6 and 12 months after surgery, including neurological examinations and cervical spine X-ray, CT and MRI examinations. The VAS and JOA score data and modified Macnab criteria results were recorded at each follow-up period.

## Results

**Fig. 4:**
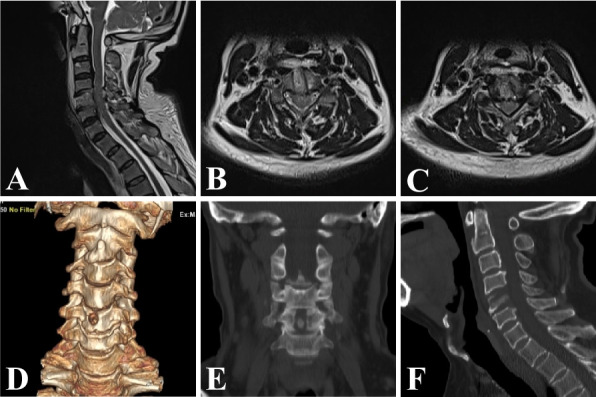
One week after surgery, sagittal and axial MRI images showed significant decompression (**A-C**). The cervical CT-3D reconstruction and coronal images showed the clear position of the entrance and two bony channels (**D**,** E**), and the CT sagittal image showed that the grafted autologous bone was complete and most of the osteophyte was removed (**F**)

**Fig. 5:**
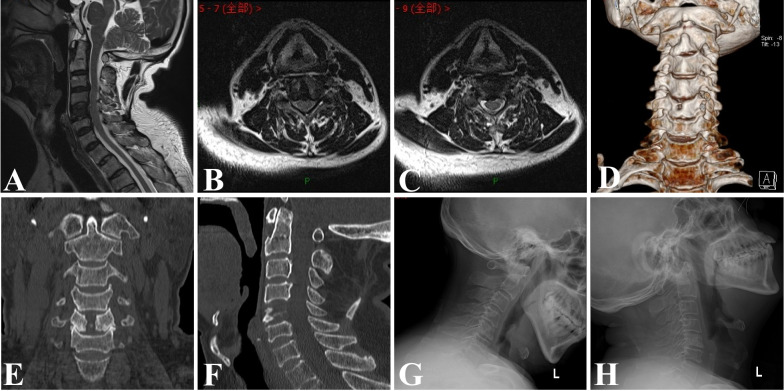
At 6 months after surgery, sagittal and axial MRI showed adequate decompression of the spinal cord (**A-C**). The CT-3D reconstruction and coronal images showed good healing of the bony channels without collapsed fractures of the drilled vertebral body (**D, E**), and no displacement of the autologous bone was observed in the sagittal position (**F**). The power position X-ray showed no loss of vertebral height or cervical instability (**G**,** H**)

 All 12 patients completed the operation successfully, with an average time of 204.83 ± 11.98 min (180–220 min) and an average intraoperative bleeding of approximately 13.56 ± 5.58 ml (6–24 ml). The general condition and clinical data of the patients are shown in Table 1. All patients were able to get out of bed 1 day after surgery and were discharged 3 to 4 days after surgery, and no postoperative complications, such as dysphagia, Horner’s syndrome, or laryngeal recurrent nerve palsy, were found. Changes in the VAS and JOA scores of patients during the postoperative periods are shown in Table 2. As shown by the analyzed data, the postoperative VAS score was significantly lower than the preoperative score (*P* < 0.001), and the postoperative JOA score was significantly higher than the preoperative score (*P* < 0.001). The improvement in VAS and JOA scores was more obvious at 6 months postoperatively than at 1 week postoperatively (*P* < 0.001), but the difference between VAS and JOA scores at 12 months postoperatively compared to 6 months postoperatively was not statistically significant (*P* > 0.05). According to the modified Macnab criteria, the clinical outcome was excellent in 8 cases, good in 3 cases and fine in 1 case at the final follow-up and the excellent and good rate was 91.7% (Table 3). Postoperative MRI images showed adequate decompression of the spinal cord (Fig. 4). Follow-up period CT and power position X-ray images showed good healing of the bony channels, no collapsed fractures of the operated vertebra, and no cervical instability (Fig. 5).

**Table 1:** The general condition and clinical data of patients (*n* = 12)

Cases	Age(years)	Gender	Diseased segment	Duration of symptom (weeks)	Follow up period (months)
1	48	M	C4/5, C5/6	15	24
2	42	M	C5/6, C6/7	13	18
3	40	M	C4/5, C5/6	9	24
4	54	F	C3/4, C4/5	12	18
5	56	F	C4/5, C5/6	30	24
6	44	M	C5/6, C6/7	18	23
7	47	F	C4/5, C5/6	16	18
8	51	M	C3/4, C4/5	18	20
9	53	F	C5/6, C6/7	22	14
10	46	M	C5/6, C6/7	17	14
11	49	F	C4/5, C5/6	19	12
12	53	M	C4/5, C5/6	24	12
Average	48.58			17.75	18.41

**Table 2:** Changes in VAS and JOA scores of patients during the postoperative periods (*n* = 12, x¯±s)

Items	Pre-o	Post-o 1 week	Post-o 6 months	Post-o 12 months
VAS	6.04 ± 0.26	2.62 ± 0.51	1.09 ± 0.22	0.56 ± 0.32
JOA	7.78 ± 1.17	12.56 ± 1.14	14.78 ± 1.15	15.62 ± 1.12

**Table 3:** Modified macnab criteria of patients (*n* = 12)

Grading	Number of cases	Performance
Excellent	8	Symptoms disappear completely and return to work and life as before
Good	3	Slight symptoms, mild limitation of activities, no impact on work and life
Fine	1	Symptoms are reduced, activities are limited, affecting normal work and life
Bad	0	No difference between before and after treatment, or even aggravation

## Discussion

For the surgical treatment of adjacent two-segment CSM, the most widely used surgical procedures internationally are the anterior open approach, including two-segment ACDF, and single-segment ACCF. Both procedures allow direct resection of herniated discs, osteophytes and ossified ligaments and restoration of intervertebral height by implantation of internal fixators, which can restore the physiological curvature of the cervical vertebra to a certain extent [14, 15]. However, when the lesion involves two segments, there is a risk of inadequate decompression with the anterior open approach. Additionally, the rate of joint nonfusion increases with the number of operated segments due to the occurrence of implant-host interface reactions [16–18], affecting the final surgical outcome. Both procedures have been found to alter the stability of the biomechanical structure of the cervical vertebra, leading to complications such as accelerated degeneration of adjacent segments, dysphagia, Horner’s syndrome, and paralysis of the recurrent laryngeal nerve [19–23], which is detrimental to the patient’s late neurological recovery.

Compared with open operations such as ACDF and ACCF, this procedure has certain advantages. The anterior open surgical incision is relatively large, and when the field of vision is exposed, the pulling force on the anterior soft tissue of the neck is large, increasing the risk of soft tissue injury. The surgical incision of this technology is only 7 mm, and the soft tissue channel is established under C-arm fluoroscopy by the “two-finger method”, which reduces pulling on the anterior soft tissue of the neck and avoids damage to the esophagus, blood vessels, nerves and other tissues. In addition, open surgery carries the risk of inadequate decompression, and the illumination equipment, magnification, and continuous saline irrigation of the spinal endoscopic system provide sufficient light to the surgical area with a clearer surgical field of view, reducing the risk of inadequate spinal cord decompression [24, 25]. This procedure restores the anatomic integrity of the cervical spine through total endoscopy combined with bone grafting through the channel, without the need to implant internal fixation, maintaining the biological structure stability of the cervical spine, and avoiding complications related to fusion surgery such as adjacent segment degeneration and secondary surgery after loosening of the internal fixation. The anterior full-endoscopic transcorporeal approach has been applied successfully in the treatment of cervical disc herniation (CDH) and single-segment CSM without the aforementioned open surgical complications, and relatively promising results have been achieved, confirming that this procedure is an effective and safe minimally invasive surgery [7–13]. On this basis, we applied APFETDSC via one vertebra with two bony channels to the treatment of adjacent two-segment CSM.

There are two main critical steps in this procedure. First, the anterior cervical safety puncture zone was established. The establishment of the anterior cervical safety zone relies on the “two-finger method.“ After marking the body position of the target vertebral body under C-arm fluoroscopy, the surgeon pushed the visceral fascia sheath medially and the carotid sheath laterally. The position of the K-wire insertion was adjusted by C-arm fluoroscopy of the front and side views to establish soft tissue access. The procedure is risky and can damage tissues such as the esophagus, carotid artery, and nerves with carelessness, requiring the surgeon to be proficient in anterior cervical anatomy. Second, two bony channels were established in one vertebral body. The entrance to the bony channel should be established in the anterior centered position of the vertebral body as much as possible to reduce the medical source of injury to the longus colli muscle and cervical sympathetic trunk nerves and to reduce the risk of intraoperative bleeding and postoperative hematoma [9, 26–28]. Preoperatively, we needed to collect detailed imaging data of the patient and use preoperative CT and MRI images as a reference to clarify the sagittal and coronal parameters of the herniated disc and the location of the osteophyte and precisely simulate the direction and trajectory of the two bony channels on the X-ray frontal and lateral radiographs [11–13]. After determining the direction and trajectory of the channels, the surgeon established two oblique bony channels using the trephine under C-arm dynamic fluoroscopy. After completing spinal cord decompression of the upper segment, the working channel was slowly withdrawn to the entrance of the bony channel. Establishment of the bony channel of the lower segment was then started after it was determined that there was no active bleeding in the channel under the endoscopic illumination system; the channel was entered directly from the entrance of the original bony channel under C-arm fluoroscopy according to the preoperatively simulated channel position.

The difficulty of this procedure lies in the creation of two directional bony channels in one vertebral body using the trephine since creating the channels is relatively challenging. The two oblique bony channels are at the same entrance, and there is partial overlap at the entrance of the channel. The rotational shaking motion of the trephine to intercept the autologous bone carries the risk of internal fracture collapse of the vertebral body and requires careful manipulation under dynamic C-arm fluoroscopy. However, the most innovative feature of this procedure is that by creating two oblique bony channels in one vertebral body and decompressing two segments, we do not have to create separate bony channels in the two vertebral bodies, which minimizes the damage to the anterior cervical tissues and vertebral bodies.

We considered the question of whether the creation of two oblique bony channels through the same access portal on one vertebral body would have an impact on the biomechanical stability of the cervical spine. Some studies have demonstrated that the physiological state of the anterior transcorporeal approach procedure is closer to the normal cervical spine model, with better biomechanical stability and the ability to maintain better cervical mobility [29–31]. The results of a finite element analysis of the biomechanical stability of the C4 single bony channel showed that the diameter of the bony channel should be < 8 mm at the time of partial endplate excision [32]. The maximum diameter of both bony channels in this study was 7.5 mm, which had relatively little effect on the biomechanical stability of the operated vertebral body. According to our follow-up imaging results, the stability of the operated vertebral body was good, and no cervical instability occurred. In addition, we trimmed the intraoperatively intercepted autologous bone and implanted it into the bony channel, which preserved the integrity of the vertebral anatomy and maintained the normal physiological curvature of the cervical spine and the stability of the operated segment [8–13]. There is relatively little research evidence regarding the effect of the anterior transcorporeal approach procedure on vertebral stability, and understanding the effect of the two bony channels established in this study on the biomechanical stability of the C5 vertebral body will require further follow-up data and mechanically relevant tests.

This study is the first report of APFETDSC via one vertebra with two bony channels as an approach to providing a minimally invasive, safe, and effective treatment for patients with adjacent two-segment CSM. The procedure simultaneously resolved spinal cord compression in two adjacent segments without the need to implant an internal fixator and avoided damage to the disc tissue. Clinical symptoms improved in all patients during the follow-up period. At the last follow-up, no fracture collapse instability of the vertebral body was observed on cervical spine images, and cervical spine mobility was good, so the surgical results were satisfactory. However, there are some limitations to the procedure. First, establishing two bony channels in one vertebra is difficult and depends heavily on the operating experience of the surgeon. Furthermore, this study focused on the disc-osteophyte complex to address ventral compression of the spinal cord, and the surgical indications were relatively narrow. Its follow-up efficacy needs to be evaluated by long-term studies on a large sample of cases.

## Conclusions

This study is the first report of anterior percutaneous full-endoscopic transcorporeal decompression of the spinal cord via one vertebra with two bony channels. This procedure has the advantages of less trauma, faster recovery, fewer complications and no need to implant internal fixators. This is a minimally invasive, feasible and safe surgical procedure for patients with adjacent two-segment CSM.

## Data Availability

The datasets used and analyzed during the current study are available from the corresponding author upon reasonable request.

## Abbreviations

CSM: Cervical spondylotic myelopathy
ACDF: Anterior cervical discectomy and fusion
ACCF: Anterior cervical corpectomy and fusion
APFETDSC: Anterior percutaneous full-endoscopic transcorporeal decompression of the spinal cord
CDH: Cervical disc herniation
VAS: Visual Analog Scale
JOA: Japanese orthopedic association score
CT: Computed tomography
MRI: Magnetic resonance imaging
